# Enhancing the compression resistance of buffer cushions using an inner concave negative Poisson’s ratio structure

**DOI:** 10.1371/journal.pone.0321379

**Published:** 2025-04-04

**Authors:** Daizhou Li, Xiufen Zhang, Haibin Li

**Affiliations:** 1 College of Science, Inner Mongolia University of Technology, Hohhot, PR China; 2 Inner Mongolia Key Laboratory of Robotics and Intelligent Equipment Technology, Hohhot, PR China; 3 College of Mechanical Engineering, Inner Mongolia University of Technology, Hohhot, PR China; Guizhou University of Finance and Economics, CHINA

## Abstract

To enhance the compression resistance of the existing buffer cushions, this paper developed a novel buffer cushion with an inner concave negative Poisson’s ratio (NPR) structure. The structure parameters of buffer cushion were optimized based on orthogonal experimental design and theoretical analysis. Furthermore, the finite element models of the NPR cushion and a comparable hexagonal cushion were established. Then the quasi-static compression and dynamic impact compression simulations and compression experiments using a 3D printed model were conducted to analyze the compression resistance of the NPR cushion. The results showed that the developed NPR cushion exhibited good compressive properties under a uniform load of at most 47 MPa, and its deformation was 73.49% of the deformation of the hexagonal cushion, indicating an improvement in compressive resistance. In the simulation analysis, the stresses of the two buffer cushions in the case of dynamic impact compression were much larger than those in quasi-static compression. The consistency between simulations and experiments results validated the design’s effectiveness in improving compression resistance, offering a valuable reference for the application of NPR structures in cushion design.

## Introduction

**Fig 1:**
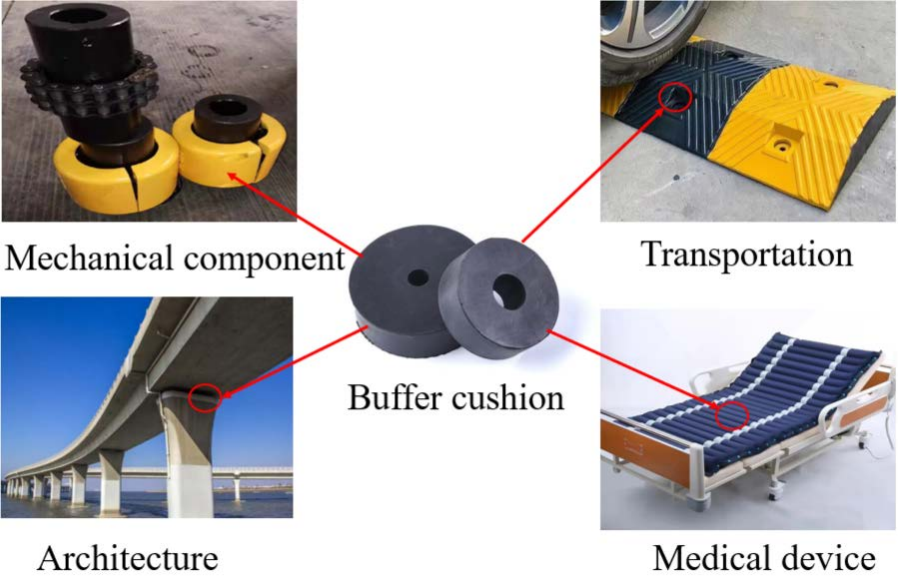
Applications of the buffer cushion.

A buffer cushion can effectively absorb the energy and reduce damage to key components, so it has a wide range of applications in machinery manufacturing, civil engineering, transportation, medical devices and other related fields [1], as shown in Fig 1. The traditional hexagonal cushion produces an anti-fragmentation or saddle curvature, when it is bent outward. Its compressive capacity and mechanical properties require further research to improve [2].

Evans et al. [3] coined the term “auxetics” to describe materials and structures with a negative Poisson’s ratio. For this type of material, when there is an external load, the deformation perpendicular to the direction of the pressure expansion, so the Poisson’s ratio is less than zero [4–6]. Structures and materials with the negative Poisson’s ratio (NPR) effect have many superior mechanical properties, including the high strength [7], impact resistance [8], energy-absorbing properties [9–11], fracture resistance [12] and sound insulation [13]. They are gradually adopted in various fields, such as civil engineering [14] and transportation [15]. As a porous medium, an NPR material has a special pore structure and properties, which are related to its negative Poisson’s ratio and affect its other mechanical properties. Therefore, in the analysis and design of porous media, the special properties of NPR materials should be fully considered [16–19].

The NPR effect of the inner concave hexagonal structure has been confirmed [20], and the study of its NPR structure has gradually received extensive attention, particularly in terms of the structural design, optimization, analysis of the mechanical properties and applications [21–23].

Studies of the mechanical properties of NPR structure, it mainly focused on their deformation mode and energy absorption capacity under impact loads. Hou et al [24] prepared aramid-fiber-augmented composite sandwich panels with cores of the conventional hexagonal structure and concave hexagonal structure, performed compression tests and obtained consistent, test results with the theory. Xiao et al [25] employed simulations and experiments to examine the structural response of an end-clamped sandwich beam featuring an auxetic reentrant hexagonal aluminum honeycomb core, which was subjected to high-velocity impact from foam projectiles. Based on the experimental findings, the failure modes of the sandwich beam were analyzed. It was observed that the sandwich beam, depending on the thickness of the honeycomb cells, could experience various failure modes. Li et al [26] prepared reentrant honeycomb cores with different cell-wall thicknesses by selective laser melting, and investigated the dynamic response of the end-clamped shallow arches of aluminum face sheets and auxetic reentrant concave hexagonal aluminum honeycomb core by simulations and experiments. Ren et al [27]proposed an auxetic tubular structure that exhibited auxetic behaviors under tensile and compressive loads; this material was used to develop a nail that could easily enter another structure but was difficult to remove. Zhu et al [28] fabricated an assembled tension-expansion structure by 3D printing and investigated its mechanical properties, which was inspired by mortise and tenon joints.

In the application of NPR structures, Leung et al [29] designed an insole with NPR characteristics based on finite element modeling, which provided more protection for the feet of diabetic patients than ordinary insoles. Jiang et al [30] designed a new intervertebral disk implant that used the NPR structure in the compression process with extraordinary energy absorption characteristics, and effectively alleviated the symptoms of lumbar intervertebral disk herniation. Wang et al [31] focused on the pedestrian protection function of systems with a negative Poisson’s ratio, which effectively protected of pedestrian legs during collisions and had high system reliability in the design of front-end buffers with multi-objective reliability optimization. Wang et al [32] developed a cylindrical buffer with a negative Poisson’s ratio and applied it to various automotive suspension structures. They found that it provided a better force-displacement curve than traditional polyurethane buffers through bump and pothole tests, which enhanced the riding comfort of vehicles.

However, most current three-dimensional negative Poisson’s ratio (NPR) materials and structures exhibit this unique property only under small strains, and many are based on pre-designed geometries. This limitation makes it challenging to modify their properties, particularly their mechanical characteristics, thereby restricting the applicability of NPR materials and structures. Existing studies have highlighted the benefits of auxetic structures and their potential applications across various fields due to their desirable properties. However, to fully realize the potential of these structures, further in-depth research is necessary to enhance their adaptability and widespread use.

Inspired by the existing research results, including those of Zhou et al [33]and Lang et al [34], based on the ideas related to the design of the inner concave NPR concave structural element, this paper designed an inner concave NPR structural buffer cushion with better compression to improve the compression property of the traditional cushion. Due to its unique geometric cross-sectional structure, this single cell structure was able to maintain high stability during deformation and was less prone to structural failure. This characteristic gave it a longer service life and reliability under cyclic loads. A hexagonal structure buffer cushion with same height and width was used as a reference. Orthogonal experimental design, theoretical analysis and ABAQUS finite element simulations were used to verify the optimal parameters of the negative Poisson’s ratio structure. The deformations of the two buffer cushions under compression were compared in finite element simulations and compression experiments.

## Methods

### Theoretical analysis of negative Poisson’s ratio structural parameters

**Fig 2:**
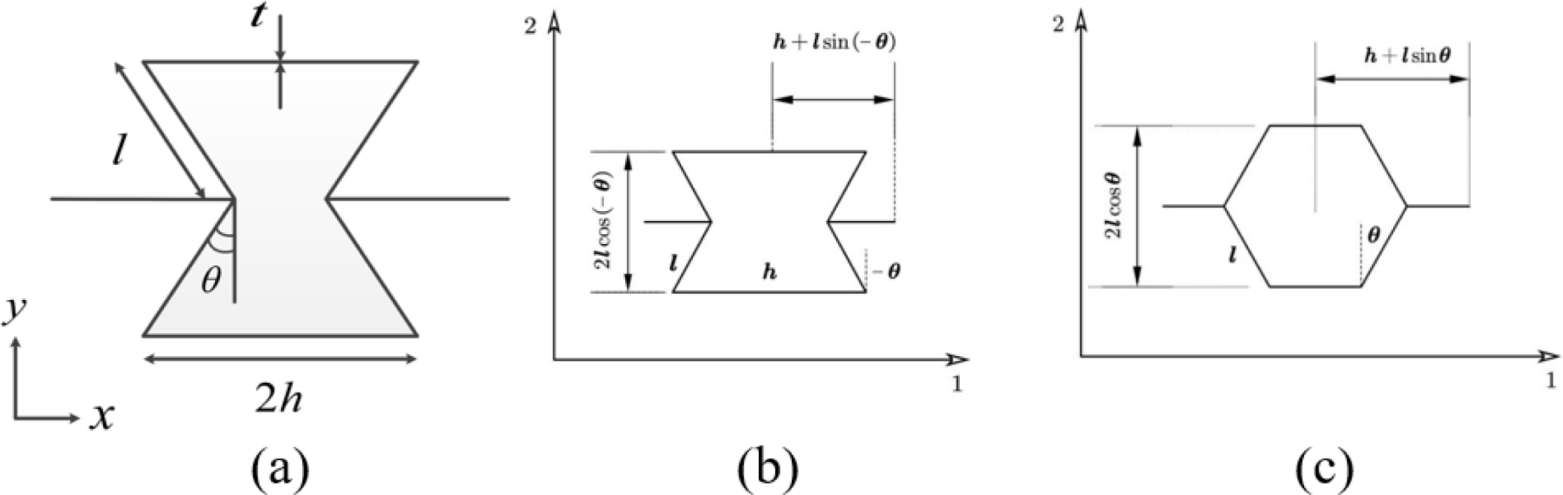
Structure of the single cell elements: **(a)** Basic structure. **(b)** Inner concave negative Poisson’s ratio cell structure. **(c)** Hexagonal cell structure [2].

An NPR structure is a type of superstructure that laterally expands when subjected to uniaxial stretching. As shown in Fig 2(a), when *θ* is positive, the single cell element is concave Fig 2(b), which exhibits NPR properties; when *θ* is negative, the single cell element is hexagonal Fig 2(c), which exhibits regular positive Poisson’s ratio properties.

The Poisson’s ratio and Young’s modulus in the direction of force are [2]:


$$\nu =\frac{\sin\theta (h/l+\sin\theta )}{\cos^{2}\theta }$$ (1)

$$E_{s}=k\frac{h/l+\sin\theta }{\cos^{3}\theta }$$ (2)

where *h* is the half-length of the horizontal prong and *l* is the side length of the diagonal prong. Parameter *k* is expressed as follows [2]:


$$k=E_{s}{\left(\frac{t}{l}\right)}^{3}$$ (3)

where *t* is the thickness of the single cell and *Es* is the Young’s modulus of the single cell material. The Poisson’s ratio and elastic modulus are two important mechanical parameters of the structure. According to the derivation process of Eqs(1)-(3), the mechanical properties are mainly determined by the configuration of structural monomers, i.e., the configuration of structural monomers can affect the NPR effect.

Eqs(1)-(3) show that to obtain the best compression resistance, *Es* should be maximized. Based on the requirements of the buffer cushions to be designed and existing research results, the structural parameters of the cell element were determined. Specifically, the half-length of the horizontal prongs should be (5–9mm), the length of the oblique prongs should be (8–12mm) and the thickness of the prong edges should be (1–3mm) were selected based on prior experimental studies and theoretical analyses [35–36]. These ranges were chosen to ensure a balance between structural stability and energy absorption efficiency, as demonstrated in similar cellular structures [37]. To further optimize the structural parameters for the best compression property, three main influencing factors *h*, *l*, and *t* were selected, and three levels were assigned to each factor according to the average equal score. Orthogonal experimental designs [38–39] were conducted, and Table 1 shows the factor levels.

**Table 1:** Orthogonal test factor level.

Factors	*h* (mm)	*l* (mm)	*t* (mm)
Level1	5	8	1
Level2	7	10	2
Level3	9	12	3

### Orthogonal experimental designs

The compression resistance (*φ*) was defined as the percentage of the height (*h*) of the single cell after compression by a force to the height before the force (*H*), as shown in Eq(4) as a test index, a suitable orthogonal table was selected for testing, and Table 2 shows the results. According to the magnitude of extreme deviation, the preferred solution was determined as A_2_B_2_C_2_. Table 3 shows the structural parameters obtained by organizing the preferred solution.

**Table 2:** The results of the orthogonal design of the experiments.

No.	*h* (mm)	*L* (mm)	*t* (mm)	*φ* (%)
1	5	8	1	97.34
2	5	10	2	97.92
3	5	12	3	96.38
4	7	8	2	99.39
5	7	10	3	99.68
6	7	12	1	95.79
7	9	8	3	97.07
8	9	10	1	96.95
9	9	12	2	96.33
K1	291.64	293.80	290.08	
K2	294.86	294.55	293.64	
K3	290.35	288.50	293.13	
k1	97.21	97.93	96.69	
k2	98.29	98.18	97.88	
k3	96.84	96.17	97.71	
R	1.45	2.01	1.19	
optimal	A_2_	B_2_	C_2_	

**Table 3:** Structural parameters of the negative Poisson’s ratio cell elements.

	*h* (mm)	*l* (mm)	*t* (mm)	*θ*
Inner concave negative Poisson’s ratio	7	10	2	-11.31°
Hexagonal	7	10	2	11.31°


$$\varphi =\frac{h}{H}$$ (4)

### Compression simulations and experiments

**Fig 3:**
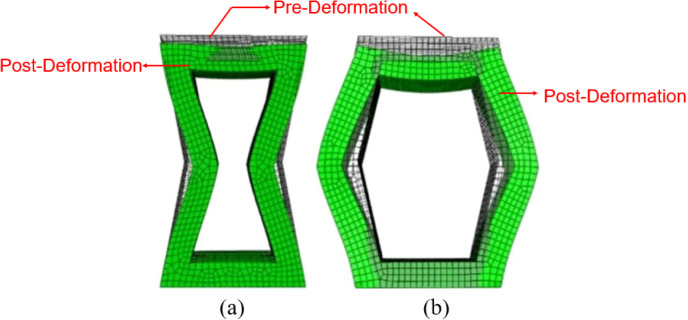
Deformation cloud of two 3D single cell structures after compression: **(a)** Inner concave negative Poisson’s ratio structure. **(b)** Hexagonal structure.

To compare the compression properties of the two single cell structures, their single cell models were constructed in ABAQUS with the parameters in Table 3, and the acrylonitrile butadiene styrene (ABS) material was used. Table 4 showed its mechanical and physical properties, and the density was set to 1.1e-9. A thickness of 5 mm was set through the stretching operation to transform the cell structure from two-dimensional to three-dimensional. Fully fixed constraints were applied to the lower face of the two single cell structures. A uniform load of 1 MPa was applied to the upper face. Fig 3. showed the analyzed results, where the green color indicated the deformed state post-deformation, and the transparent cells indicated the state of the structure pre-deformation.

**Table 4:** Mechanical and physical properties of the ABS material [40].

Mechanical properties	Physical properties
Tensile strength: 47 MPa	Hardness: 78HB
Modulus of elasticity: 2200 MPa	Melting point: 217 ~ 237°C
Poisson’s ratio: 0.394	Density: 1.02 ~ 1.18g/cm^3^
Shear modulus: 318.9 MPa	Thermal conductivity: 0.2256 W/(m-K)
Fracture strength: 40 MPa	Specific heat capacity: 1386J/(kg-K)

As can observed in Fig 3, both structures maximally deformed at the center of the upper end face, and the inner concave NPR structure had a greater compression resistance than the hexagonal structure, the reason was that the NPR structure in the location of the force occurs in the densification resulting in the existence of the pressure acting on the place of the density of the instantaneous increase in the phenomenon occured, and resistance to compression and deformation of the capacity of the enhancement of the compression, and the compression resistance was brought into full play. When subjected to compressive loading, the cells in the structure rotate and rearrange each other. This movement mainly occur during the migration of unloaded regions to loaded regions. Specifically, the geometry and connections of the cells allow them to approach each other in a unique manner when subjected to compression, resulting in an increase in density in localized regions.

**Fig 4:**
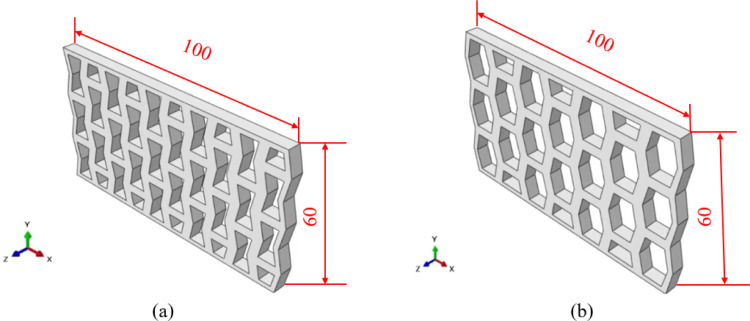
Inner concave negative Poisson’s ratio structure and hexagonal structure of multi cell elements: **(a)** Inner concave negative Poisson’s ratio structure multi cell element. **(b)** Hexagonal structure multi cell element.

To avoid the contingency of the above analysis results, the translation and linear array functions of ABAQUS were applied to construct three-dimensional multi cell models of the inner concave. Fig 4 shows NPR and hexagonal cells. The same length and width were set for the two multi cell structure models: L = 100mm; H = 60mm. The thickness of the model was 5mm, and the two multi cell structures had identical overall dimensions to enhance the persuasiveness of the simulation results. To avoid the interaction among the cell elements, the multi cell structure was set as having no boundary.

**Fig 5:**
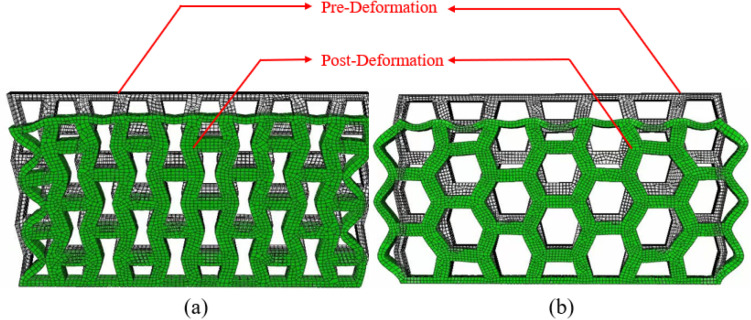
Deformation cloud maps of two multi cell structures: **(a)** Inner concave negative Poisson’s ratio structure multicellular element. **(b)** Hexagonal structure multi cell element.

The constraints and loading conditions were similar to those of the single cell model, where the fixed-end constraints were on the lower bottom surface. The distributed load was 1 MPa on the upper end surface and in the negative y-direction. Fig 5 shows the structural deformation cloud of the two 3D multi cell structural models.

As the simulation results in Fig 5 show, when the upper end faces of the two multi cell structures were subjected to a certain pressure, the hexagonal multi cell structure more strongly collapsed outward and had a serious inward deformation. In particular, the stressed portion of the upper end face (which can be called to as the central ballast area) was easily deformed. The inner concave NPR multi cell structure collapsed less inwardly on both sides when compressed, and the degree of concavity in the central region of the upper end face was smaller. The main reason was that the compression effect increased the internal density of the structure and strengthened the resistance to compression deformation. Although the overall mass and volume of the structure remains the same, due to the rearrangement of the cells, the distribution of material in certain areas becomes denser, which manifests itself as an increase in density. Thus, the structural strength improved, and more energy was required to produce a larger deformation. In other words, the structure had good stiffness and excellent energy-absorbing properties.

**Fig 6:**
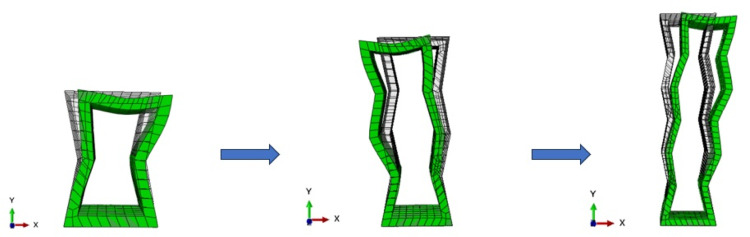
Inner concave negative Poisson’s ratio single cell considering scale effects.

Fig 6 illustrated the inner concave negative Poisson’s ratio single cell considering scale effects. Negative Poisson’s ratio material shrank laterally when subjected to compression, and this effect persisted after increasing the number of cells in the length direction, as the geometrical properties and deformation mechanism of the overall structure remain unchanged. Due to its unique geometric cross-sectional structure, this single cell structure was able to maintain high stability during deformation and was less prone to structural failure. This characteristic gave it a longer service life and reliability under cyclic loads.

### Parameterization of the buffer cushions

For cushioning materials, the long-term compression resistance of the material is an important factor. To provide long-lasting cushion and protection, the material must deform or lose its elastic effect after a long period of use. From the above simulation analysis results of a single-cell structure and a multi-cell structure, the inner concave NPR had significantly better compression resistance and energy absorption than the hexagonal structure with identical specifications. Therefore, the inner concave NPR structure was applied to the design of buffer cushions.

**Fig 7:**
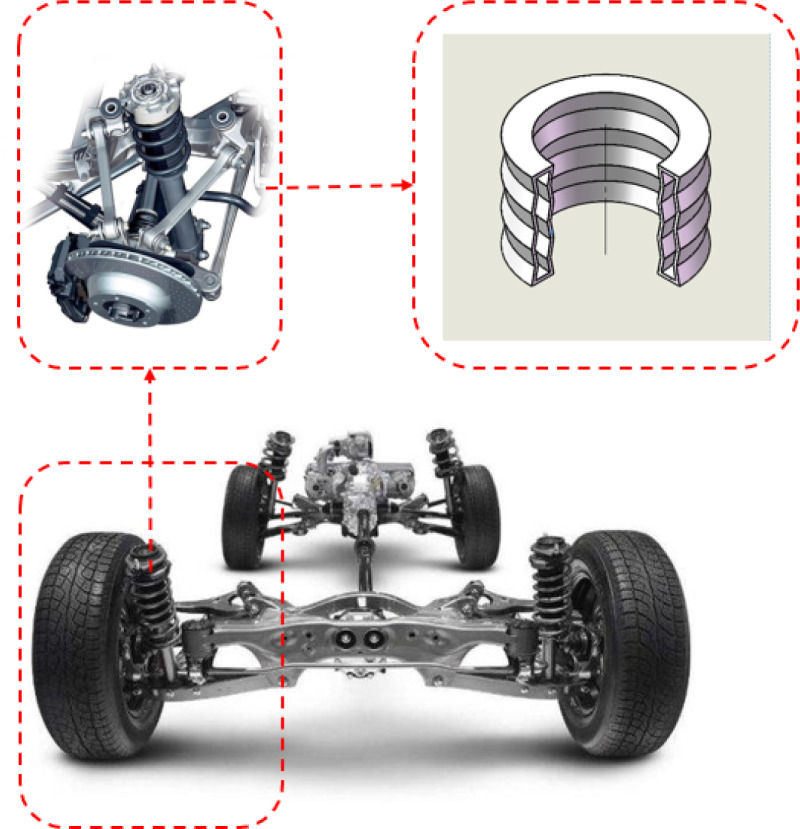
The cross-sectional view models of an inner concave negative Poisson’s ratio buffer cushion and its application scenarios.

Due to its distinctive geometric cross-sectional design, this single cell structure maintained high stability when deformed and was less likely to experience structural failure. This feature resulted in a longer service life and enhanced reliability under cyclic loading. The two buffer cushion models were designed under conditions that closely mimicked the actual working conditions considering the equivalence theory. Three layers of cell elements were superimposed in the vertical direction. The overall dimensions of the two models were as follows: inner diameter: 33mm; outer diameter: 47mm and height: 60mm. This novel type of negative Poisson’s ratio structure cushion can be used in the suspension of new energy electric vehicles, as shown in Fig 7, effectively reducing vibrations and noise caused by bumps, thereby improving the smoothness of the vehicle during operation.

## Results and disscussion

### Construction of the finite element model for buffer cushions

The SOLIDWORKS software was applied to create a three-dimensional buffer cushion model, which was imported into the finite element software ABAQUS for analysis.

Because this manuscript used simulation for structural verification, and the appearance of the model does not require excessive precision, the selection must have excellent mechanical properties and affordable materials, such as polylactic acid (PLA), acrylonitrile butadiene styrene (ABS), Acrylonitrile-Styrene-Acrylate (ASA) and other plastic materials. The ABS material has high strength, good toughness and excellent property in various fields. A series of comparisons showed that the ABS material surface was relatively flat, and commonly used to produce outdoor infrastructures and commercial structural application of parts, whereas the demand for the buffer cushion of this paper was more low-cost. Therefore, in the simulation, the thermoplastic ABS was used as the filler material of the inner concave NPR structure.

### Grid convergence analysis

**Fig 8:**
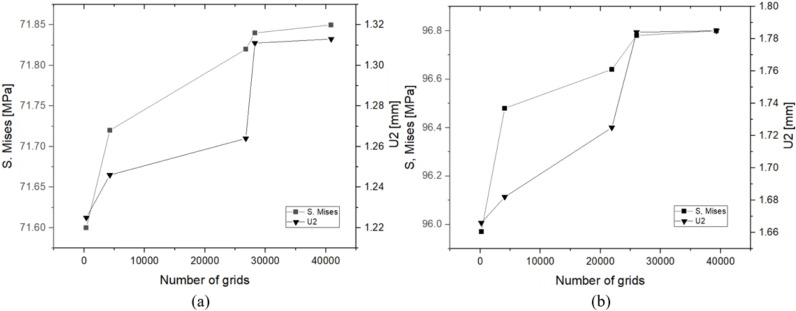
Grid convergence analysis: **(a)** Inner concave negative Poisson’s ratio buffer cushion and **(b)** Hexagonal buffer cushion.

We carried out grid convergence analyses of inner concave negative Poisson’s ratio structural cushion and hexagonal structural cushion. Through the results shown in Table 5–6 and Fig 8, the effects of different grid numbers on the S.Mises stresses and U2 displacements were gradually weakened, indicating that the grid refinement improved the accuracy of the calculation results.

**Table 5:** Grid convergence analysis of inner concave negative Poisson’s ratio structural buffer cushion.

Number of grids	S. Mises (MPa)	Error (%)	U2 (mm)	Error (%)
360	71.60		1.225	
4260	71.72	0.168	1.246	1.714
26750	71.82	0.139	1.264	1.445
28248	71.84	0.029	1.311	3.718
40866	71.85	0.014	1.313	0.153

**Table 6:** Grid convergence analysis of hexagonal structural buffer cushion.

Number of grids	S. Mises (MPa)	Error (%)	U2 (mm)	Error (%)
180	95.97		1.666	
4080	96.48	0.531	1.682	0.960
21888	96.64	0.166	1.725	2.556
26015	96.78	0.145	1.784	3.420
39288	96.80	0.021	1.785	0.056

Specifically, in the inner concave negative Poisson’s ratio structure, the S.Mises stress and U2 displacement were close to stabilized when the number of grids reaches 40866, and the errors were reduced to 0.014% and 0.153%, respectively. Whereas in the hexagonal structure, the errors in S. Mises and U2 had been reduced to 0.021% and 0.056% for a grid number of 39288.

Due to the complexity of the part model, this paper used the common C3D8R solid cells for intelligent mesh delineation to reduce the global size and refine the mesh in the stressed parts.

### Quasi-static compression simulations and experiments

Quasi-static compression simulations were performed for the inner concave NPR structure buffer cushion and hexagonal buffer cushion. The upper surface of the two types of buffer cushion was compressed using a homogeneous load in negative y-direction. The loading rate for the compression simulation is 0.1mm per minute. The homogeneous load size gradually increased from 0 to 47 MPa, which was the maximal tensile strength of the material.

**Fig 9:**
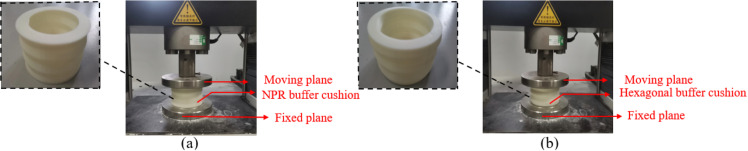
Compression experiments of buffer cushions: **(a)** Inner concave negative Poisson’s ratio structure. **(b)** Hexagonal structure.

The two buffer cushions were printed using a Raise Pro 2 3D printer with ABS consumables as the raw materials. The compression experiments were conducted in a controlled laboratory environment with a constant temperature of 23 ±  1°C and relative humidity of 50 ±  5%. These conditions were maintained throughout the testing period to ensure consistency in material behavior. Two different shapes of buffer cushions were compressed under identical experimental conditions using a universal testing machine with an acceleration rate of 0.1 mm/s² (Fig 9). The universal testing machine can test the mechanical properties of materials such as the strength and stiffness.

Through the quasi-static compression simulations and quasi-static compression experiments of the universal testing machine, the compression resistance of the two cushions was effectively measured based on the previously defined compression resistance index. The compression experiments were repeated three times to ensure data reliability. The mean values of their deformations, along with their standard deviations, are summarized in Table 7. Table 7 presented the statistical data from compression experiments, including the results from three experiments (EXP1, EXP2, and EXP3) for two different cushion types: Inner concave negative and Hexagon. For the Inner concave negative Hexagon, the mean deformation was 1.311 mm with a standard deviation of 0.0037 mm², and the average φ was 97.82%. For the second cushion type, the mean deformation was 1.784 mm with a standard deviation of 0.0093 mm², and the average φ was 97.03%. These results indicated consistent performance across the repeated experiments.

**Table 7:** Compression experiments related statistical data.

	EXP1	*φ* (%)	EXP2	*φ* (%)	EXP3	*φ* (%)	Mean values (mm)	*φ* (%)	Standard deviations (mm^2^)
Inner concave negative	1.310	97.817	1.307	97.822	1.316	97.807	1.311	97.82%	0.0037
Hexagonal	1.777	97.038	1.785	97.025	1.790	97.017	1.784	97.03	0.0093

**Fig 10:**
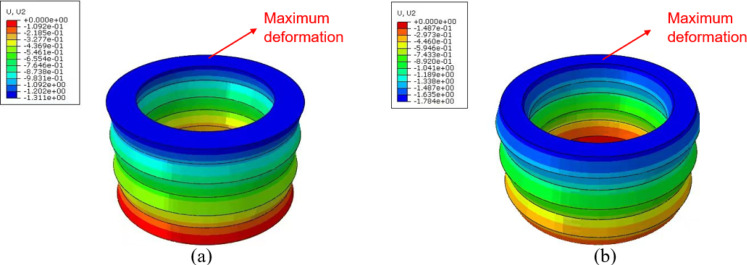
Comparison of the displacements in the y-direction for the **(a)** Inner concave negative Poisson’s ratio structural buffer cushion and **(b)** Hexagonal structural buffer cushion.

**Fig 11:**
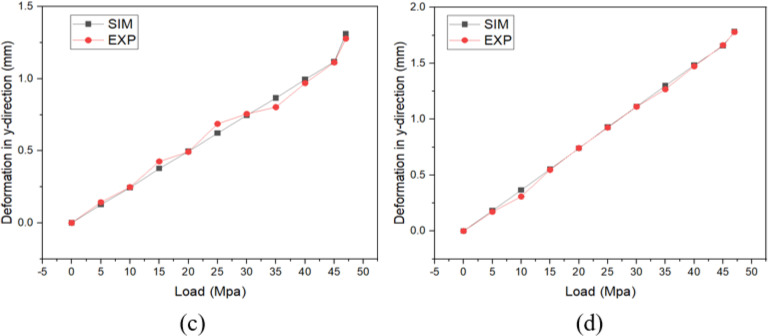
Comparison curves between simulation and experiment for the **(a)** Inner concave negative Poisson’s ratio structural buffer cushion and **(b)** Hexagonal structural buffer cushion.

In Fig 10, the inner concave NPR buffer cushion showed a significantly smaller and more uniform deformation than the hexagonal buffer cushion, which indicated its better stability. Fig 10(a) and (b). compared the displacements in the y-direction of between the inner concave NPR structural cushion and hexagonal cushion, respectively. In Fig 11(a) and (b), the mean deformation of the inner concave NPR structure buffer cushion was 1.311 mm, and the mean compression resistance was calculated to be 97.82%. Furthermore, the mean deformation of the hexagonal structure buffer cushion was 1.784 mm, and the mean compression resistance was 97.03%. Thus, the deformation of the former cushion was 73.49% of that of the latter, and the inner concave NPR structure exhibited better compression resistance than the traditional hexagonal structure, i.e., it had better compression property.

### Dynamic impact compression simulations

Dynamic impact compression simulations were usually achieved by applying velocity or displacement. In ABAQUS, these can be simulated by using the “Dynamic, Explicit” type in the analysis step. A velocity of 10 m/s was applied to the two buffer cushions at the upper face, in a direction perpendicular to the upper face, restricting all degrees of freedom except the vertical direction.

**Fig 12:**
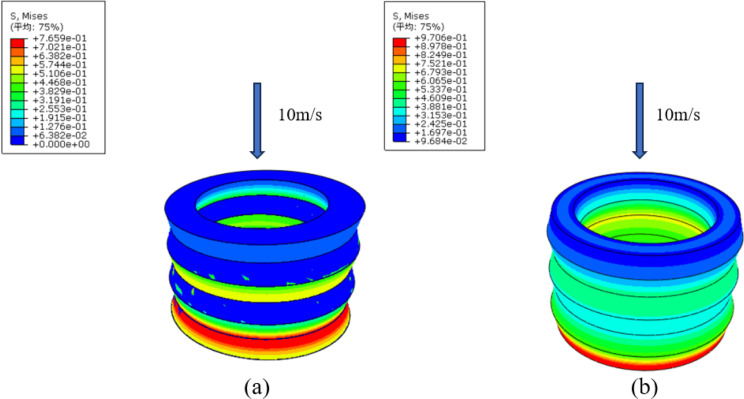
Dynamic impact stress contour plots of the **(a)** Inner concave negative Poisson’s ratio structural buffer cushion. **(b)**Hexagonal structural buffer cushion.

Fig 12 showed the dynamic impact stress contour plots of the inner concave negative Poisson’ ratio structural buffer cushion and hexagonal structure buffer cushion. Two buffer cushions were subjected to an impact speed of 10 m/s. Dynamic impact results in higher stress concentration and quicker response times. The inner concave negative Poisson’s ratio buffer cushion might demonstrate more distributed stress under quasi-static conditions, while the hexagonal structure might retain its uniform stress pattern but with less intensity. These observations suggested that under dynamic impact conditions, stress localization was more pronounced compared to quasi-static compression experiments, where stress spread more evenly across the structures.

The focus of this paper was mainly on quasi-static compression experiments. There was that we wished to gain an in-depth understanding of the mechanical behavior of materials under this particular stress state through detailed analysis under quasi-static conditions. Based on the results of our analyses so far, we had found that quasi-static compression experiments had been sufficient to reveal the key properties of the materials and provide adequate support for our research objectives. Due to the limitation of experimental conditions, only dynamic impact simulations were performed.

## Conclusions

In this paper, a novel inner concave negative Poisson’s ratio buffer cushion was designed and its compression resistance was studied. The inner concave negative Poisson’s ratio cell structure parameters were optimized based on the orthogonal experimental design, theoretical analysis and ABAQUS finite element simulations. Then the simulations and compression experiments using a 3D-printed model were conducted to analyze the compression property. The following conclusions were obtained:

(1) A novel inner concave negative Poisson’s ratio buffer cushion was designed based on the orthogonal experimental designs, which reduced the number of experiments, effectively compared the experiment results and found the optimal structure parameters. This novel type of negative Poisson’s ratio structure cushion can be used in the suspension of new energy electric vehicles, effectively reducing vibrations and noise caused by bumps, thereby improving the smoothness of the vehicle during operation.
(2) The buffer cushion’s compression property was studied by simulation analysis, and the research results showed that two types of buffer cushion under uniform load size of 47 Mpa, inner concave NPR structure buffer cushion had a deformation of 1.311 mm and a compression resistance of 97.82%. The hexagonal structure buffer cushion had a deformation of 1.784 mm and a compression resistance of 97.03%. Under the same compressive load, the deformation of the concave NPR structure is 73.49% of that of traditional hexagonal structures, indicating an improvement in compressive resistance. The experimental results aligned closely with the simulation findings.
(3) In the simulation analysis, the stresses of the two cushions in the case of dynamic impact compression were much larger than those in quasi-static compression, under dynamic conditions, stress localization was more pronounced compared to quasi-compression, where stress spread more evenly across the structures and the stresses of the inner concave negative Poisson’s ratio structural cushion was smaller than those of the hexagonal structural cushion.

These conclusions can provide valuable insights for the design and application of inner concave NPR structures. However, the high costs and low production efficiency associated with 3D printing technology continue to pose significant challenges for the engineering applications of these structures. Moving forward, we aim to explore more efficient additive manufacturing methods to investigate the mechanical properties of the inner concave NPR structure cushions, including fracture resistance, energy absorption, heat transfer characteristics, fatigue life, and thermal conductivity. This broader investigation will ensure a more comprehensive understanding of the structures’ performance in practical applications.

## Supporting information

S1 File The supporting data file for Fig 8(a). (ZIP)

S2 File The supporting data file for Fig 8(b). (ZIP)

S3 File The supporting data file for Fig 11(a). (ZIP)

S4 File The supporting data file for Fig 11(b). (ZIP)
